# Hypoxia as a barrier to immunotherapy in pancreatic adenocarcinoma

**DOI:** 10.1186/s40169-019-0226-9

**Published:** 2019-04-01

**Authors:** S. K. Daniel, K. M. Sullivan, K. P. Labadie, V. G. Pillarisetty

**Affiliations:** Department of Surgery, University of Washington, Seattle, USA

**Keywords:** Hypoxia, Immunotherapy, Solid tumor, Pancreatic cancer

## Abstract

Pancreatic ductal adenocarcinoma (PDA) is a lethal disease with limited response to cytotoxic chemoradiotherapy, as well as newer immunotherapies. The PDA tumor microenvironment contains infiltrating immune cells including cytotoxic T cells; however, there is an overall immunosuppressive milieu. Hypoxia is a known element of the solid tumor microenvironment and may promote tumor survival. Through various mechanisms including, but not limited to, those mediated by HIF-1α, hypoxia also leads to increased tumor proliferation and metabolic changes. Furthermore, epithelial to mesenchymal transition is promoted through several pathways, including NOTCH and c-MET, regulated by hypoxia. Hypoxia-promoted changes also contribute to the immunosuppressive phenotype seen in many different cell types within the microenvironment and thereby may inhibit an effective immune system response to PDA. Pancreatic stellate cells (PSCs) and myofibroblasts appear to contribute to the recruitment of myeloid derived suppressor cells (MDSCs) and B cells in PDA via cytokines increased due to hypoxia. PSCs also increase collagen secretion in response to HIF-1α, which promotes a fibrotic stroma that alters T cell homing and migration. In hypoxic environments, B cells contribute to cytotoxic T cell exhaustion and produce chemokines to attract more immunosuppressive regulatory T cells. MDSCs inhibit T cell metabolism by hoarding key amino acids, modulate T cell homing by cleaving L-selectin, and prevent T cell activation by increasing PD-L1 expression. Immunosuppressive M2 phenotype macrophages promote T cell anergy via increased nitric oxide (NO) and decreased arginine in hypoxia. Increased numbers of regulatory T cells are seen in hypoxia which prevent effector T cell activation through cytokine production and increased CTLA-4. Effective immunotherapy for pancreatic adenocarcinoma and other solid tumors will need to help counteract the immunosuppressive nature of hypoxia-induced changes in the tumor microenvironment. Promising studies will look at combination therapies involving checkpoint inhibitors, chemokine inhibitors, and possible targeting of hypoxia. While no model is perfect, assuring that models incorporate the effects of hypoxia on cancer cells, stromal cells, and effector immune cells will be crucial in developing successful therapies.

## Background

Pancreatic ductal adenocarcinoma (PDA) is projected to be the second highest cause of death from cancer in the United States within the next 10 years [1, 2]. The lethality of the disease is in part due to lack of effective screening resulting in later stage diagnoses, as well as poor response to standard therapies including surgery, systemic chemotherapy, and external beam radiation [3–6]. Immunotherapy has heralded a new era in oncologic treatment that may ultimately improve outcomes, while having fewer toxic side effects than systemic chemotherapy. The overarching goal of immunotherapy is to enhance the body’s immune response to tumor cells. The strategy of blocking immune checkpoints to potentiate immune-mediated tumor cell killing has been successful in several tumors such as melanoma and certain phenotypes of lung cancer, but has not been successful in many other solid tumors such as PDA [7–9].

The reason for the effectiveness of immunotherapy in some tumors more than others has been a subject of intense focus. Initially, this was thought to be due to a paucity of immune cells infiltrating PDA tumors, however many studies have since shown there is a variable but substantial population of tumor-infiltrating lymphocytes (TIL) in PDA [10–12]. Another theory was that PDA was not as immunogenic as other tumors, but several neoepitopes have been identified as recognizable by T cells [13]. PDA in particular has a robust tumor microenvironment composed of myofibroblasts and immune cells that often outnumber carcinoma cells [12]. The interactions among these cells are undoubtedly a major driving factor of immunotherapy resistance in PDA, but hypoxia has an underlying influence that is not yet fully understood.

The tumor microenvironments of many solid tumors are known to be hypoxic [14–16]. In PDA, there is a decrease in tissue partial oxygen pressure in tumors, with median pO_2_ 0–5.3 mmHg (0-0.7%) compared to nearby normal pancreas at pO_2_ 24.3–92.7 mmHg (3.2–12.3%) [17]. For reference, normal pO_2_ is 160 mmHg (21.1%) in air and 100 mmHg (13.2%) in arterial blood [18]. Further studies have shown that this hypoxia is heterogeneous throughout the tumor and not static [17, 19, 20]. Many reviews have summarized in general the pro-survival and pro-metastatic changes that a tumor undergoes in a hypoxic environment [21–25]. Additionally, hypoxia also induces changes in the other cells in the tumor microenvironment that encourage immunosuppression, which may play a role in diminishing the efficacy of immunotherapy in PDA.

## Signaling pathways in response to hypoxia

A large number of downstream effects of hypoxia are mediated by a transcription factor called hypoxia inducible factor (HIF) [23]. Three variants of the alpha subunit of HIF have been discovered, with HIF-1α being the most commonly studied. Based on current knowledge, HIF-3α primarily acts to promote or inhibit other HIF complexes [21]. The HIF variants are constitutively expressed proteins. Primary regulation is achieved by hydroxylation of a proline in normoxic conditions by a prolyl hydroxylase unique to each HIFα variant [26]. The hydroxyl group tags the molecule for degradation via von Hippel-Lindau protein (vHL). In hypoxic conditions, the iron atom in prolyl hydroxylase stays reduced and the enzyme is unable to add the hydroxyl group to the HIFα unit [26]. This allows HIF-1α to bind to the HIF-1β molecule and translocate to the nucleus where it acts as a transcription factor on many promoter sequences.

Post-translational modifications, such as phosphorylation and acetylation, of the different variants influence binding abilities and therefore transcriptional effects [21]. Additionally, owing to their different prolyl hydroxylases, HIF-1α and HIF-2α accumulate at different oxygen levels. HIF-2α tends to accumulate at higher oxygen levels (2–5%), whereas HIF-1α does not accumulate until lower oxygen levels (0–2%) [26]. HIF-1α mRNA also degrades very quickly even in hypoxic conditions making its effects shorter lived [27]. While HIF-2α has been studied less than HIF-1α, there have been data to show that HIF-2α uniquely promotes chronic pancreatitis in mouse models, as well as mucinous cyst neoplasms in the presence of an oncogenic KRAS mutation [28].

## Hypoxia promotes tumorigenesis in carcinoma cells

Tumor cells in PDA have many advantages under hypoxic conditions. KRAS mutations, which are seen in around 95% of PDA tumors, work to alter the cell metabolism to function in hypoxic environments [29]. Glycolysis becomes the primary means of obtaining energy via downstream effects of HIF-1α and persists even if normoxic conditions are restored—a phenomenon known as the Warburg hypothesis [29, 30]. To increase the glucose supply, HIF-1α mediates increased transcription of GLUT1 and GLUT3 transporters, as well as increased production of pyruvate and lactate dehydrogenase [31, 32]. The lactate produced from glycolysis is further used as energy for surrounding cells and impairs T cell cytokine production [33]. Other metabolic enzymes that are upregulated by PDA in hypoxia include carbonic anhydrase and indoleamine 2,3 dioxygenase (IDO), which also impair immune cell function through the creation of acidic and tryptophan-depleted environments, respectively [32, 34–36]. An additional source of energy for PDA cells is autophagy, which in tumors with loss of p53 has been correlated with increased tumor progression [37–39]. Hypoxia has been shown to increase autophagy via HIF-1α, which promotes survival of PDA tumor cells, particularly those that are undifferentiated [40–42].

Mechanisms that promote terminal cell differentiation are inhibited via interaction with HIF-1α and NOTCH signaling in PDA and other tumor types [43–45]. This is thought to promote cancer cell “stemness” in hypoxic niches in the PDA microenvironment [46, 47]. The quiescent cell is also less affected by systemic chemotherapy and radiotherapy that acts on rapidly dividing cells, therefore promoting tumor recurrence [48–50]. Additional mechanisms of cell cycle regulation in tumors involve the differential expression of HIF-1α and HIF-2α. As mentioned earlier, extreme hypoxia promotes HIF-1α stabilization and actually decreases cellular proliferation by halting the cell cycle via c-MYC in some tumors [51]. Molecular inhibitors of c-MYC halt cell cycle progress in PDA and may also block hypoxic signaling [52–54]. Evidence supports that, conversely, HIF-2α promotes proliferation with cells entering the cell cycle via stabilization of MYC and increased DNA repair enzymes [25, 27].

Another benefit of a hypoxic environment for PDA is an increase in cell migration. There are numerous pathways activated by HIF-1α that contribute to the epithelial to mesenchymal transition (EMT) [55–57]. EMT involves loss of normal cell-to-cell adhesion molecules seen in terminally differentiated epithelial cells and promotes molecules used for cell movement and angioinvasion typically seen in less differentiated cells. Well documented molecules that are increased in hypoxic PDA include matrix metalloproteinases (MMPs) via up regulated fascin and QSOX1 that subsequently decrease the immediately surrounding extracellular matrix (ECM) to allow tumor cell movement [58, 59]. Cadherins are cell adhesion molecules involved in maintaining epithelial tissue architecture, with increased N-cadherin and loss of E-cadherin expression associated with greater invasive potential in cancer. Through HIF-1α-mediated NF-κB pathways, hypoxia increases N-cadherin to allow transendothelial migration into blood vessels [60]. The transcription factors snail and slug, also HIF-1α promoted, are expressed in many pancreatic cancer cell lines and act to decrease E-cadherin seen in normal epithelial cell-to-cell adhesion [61]. Twist is another transcription factor that prevents E-cadherin formation as well as increased vascular endothelial growth factor (VEGF)-A, but is primarily up regulated in PDA after HIF-2α stabilization [61–63]. Hedgehog signaling in PDA, which is potentiated by hypoxia, also down regulates E-cadherin and up regulates vimentin, which promotes invadopodia formation and angioinvasion [64].

Tumor cell survival in hypoxia also requires changes to avoid internal mechanisms of apoptosis and increase resistance to chemotherapy. In PDA cell lines, HIF-2α up regulates survivin production, which provides resistance to apoptosis by tumor necrosis factor (TNF) related apoptosis inducing ligand (TRAIL) [65]. Hypoglycemia-mediated apoptosis is also prevented in PDA by hypoxia-induced up regulation of asparagine synthetase and subsequent prevention of c-jun-NH2 terminal kinase/phospho-stress-activated protein kinase activation, which is also a method for cisplatin resistance [66]. Many pancreatic cell lines down-regulate expression of BNIP3, which is a gene involved in hypoxia-mediated cell-induced apoptosis [67, 68]. Additionally, loss of BNIP3 expression in hypoxia has been associated with resistance to gemcitabine and 5-fluorouracil [69]. Gemcitabine resistance is also increased in hypoxia via the PI3K/Akt/NF-κB pathways that increases anti-apoptotic proteins such as Bcl-XL, FLIP, and cIAP [4, 46, 70]. Hypoxia also promotes resistance to radiotherapy through decreased production of DNA free radicals and increased DNA repair enzymes as described above [46, 71].

Hypoxia induces several changes on the cell surface of tumors to promote cell survival. While not yet shown in PDA, hypoxia in prostate cancer encourages tumor cells to shed their major histocompatibility complex (MHC) class I molecules via decreased nitric oxide (NO) and increased matrix metalloproteinases (MMPs) [72]. There are increased soluble levels of MHC class I chains A and B, suggesting that PDA may use this mechanism to avoid recognition by adaptive and innate immune mechanisms [73]. Additionally, human leukocyte antigen G (HLA-G) is a component of MHC class I expressed in a minority of PDA tumors that induces immunosuppression by interacting with receptors on antigen presenting cells [74]. The up regulation of HLA-G transcription is also mediated by HIF-1α, however some other types of tumors actually have decreased HLA-G expression in hypoxia [35]. Hypoxia also promotes increased programmed death ligand-1 (PD-L1) cell surface expression in a variety of solid tumors via the PTEN/PI3K pathway through HIF-1α [72]. Increased PD-L1 expression prevents effector T cells from initiating apoptosis of cancer cells and can actually lead to anergy or apoptosis of the T cells [72]. A minority of human PDA samples with baseline PD-L1 upregulation were also seen to have downregulation of MHC class I [75]. Hypoxia also stimulated CD47 expression in PDA, which is a co-stimulatory molecule that blocks pro-phagocytic signals in myeloid derived suppressor cells (MDSCs) and macrophages [76, 77]. Tumor cells also increase CD39 and CD73 in response to hypoxia, which promotes extracellular adenosine accumulation and can lead to T cell apoptosis [78, 79].

## The effects of hypoxia on the tumor microenvironment

As discussed above, carcinoma cells themselves respond to hypoxia in self-promoting ways and encourage a continued hypoxic and acidic environment within the tumor stroma. The resulting landscape causes non-carcinoma tumor cells to shift towards an overall tumor-supportive and immunosuppressive milieu. The resulting cell phenotypes in the microenvironment have a direct influence on effector T cell function and resulting ineffectiveness of immunotherapies.

## Pancreatic stellate cells and fibroblasts

Major contributors of the tumor microenvironment are activated pancreatic stellate cells (PSCs) and myofibroblasts. PSCs are identified as having large vitamin A droplets in their inactivated state, which they lose when they become differentiated in response to pancreatic injury or inflammation [80]. Cytokines shown to activate PSCs include TGF-β, TNF-α, IL-1, and IL-6; however, some suggest they are capable of autocrine signaling [81, 82]. The role and origin of PSCs are still not fully elucidated; however, they produce ECM molecules such as alpha smooth muscle actin (aSMA), type I collagen, fibronectin, and periostin that lead to pancreatic fibrosis [81, 83]. There are differences between PSCs and fibroblasts such as cell shape, amount of different ECM molecules produced, and scavenger receptors, but often they are grouped together in discussions [82]. In pancreatic intraductal neoplasms (PanIN), which are precursor lesions to PDA, fibroblasts show a CD34^+^ aSMA^−^ phenotype, whereas in PDA, these are reversed to CD34^−^ aSMA^+^, demonstrating an increase in aSMA production as the lesion progresses from non-invasive to invasive [84]. Activated fibroblasts in PDA can often be identified by the serine protease fibroblast activating protein (FAP) expression, although this is also seen on some tumor cells, and has been associated with increased desmoplasia and worse prognosis [85, 86].

PSCs have a significant role as potentiators of immunosuppression in PDA. Increased type I collagen density produced in fibrosis interferes with chemokines used in T cell homing causing them to become “trapped” away from tumor cells. Fibroblasts also produce increased CXCL12 which is another method of inhibiting T cell homing [84, 87]. Fibronectin deposition in the ECM encourages more rapid migration of tumor cells [86]. Periostin increases fibroblast growth factor (FGF) 2 which promotes macrophage differentiation into the M2 phenotype as well as encourages PDA proliferation [88]. Cytokines produced by PSCs also exert significant influence on the tumor-infiltrating immune cells. The most impactful of this is secretion of IL-6 and M-CSF which recruits MDSCs [89, 90]. They also secrete IL-1 and TGF-β which work to continually activate PSCs to continue forming a fibrotic environment in an autocrine fashion [80].

In response to hypoxia, tumor cells produce the sonic hedgehog ligand which acts in a paracrine manner on myofibroblasts by binding to the Patched-1 receptor. The resulting downstream effects of this interaction include increased desmoplasia with production of aSMA, type I collagen, fibronectin, and periostin [91, 92]. Like in tumor cells, hypoxia up regulates carbonic anhydrase and GLUT1 and GLUT3 transporters in PSCs to further contribute to the immunosuppressive microenvironment. Additionally, hypoxia promotes connective tissue growth factor production by PSCs, which helps inhibit apoptosis in tumor cells [93]. VEGF production from PSCs is also increased in hypoxic conditions, which works in a paracrine fashion to encourage PSC migration [91, 94, 95].

## Plasma or B cells

Responsible for the humoral immune response, B cells were once thought to reside primarily in lymphoid tissue, but studies have recently shown that they also infiltrate the tumor microenvironment [96]. CXCL13, a primary chemokine for B cell migration, is expressed by fibroblasts in the PDA stroma [97]. While not as extensively studied as other immune cell populations, there have been conflicting data about the role of B cells in the anti-tumor response. It has been elucidated that B cells, much like the rest of tumor-infiltrating immune cells, exist on a spectrum of activation that can encourage or inhibit T cell responses [96, 98]. One study reported that IL-35-producing B cells stimulate pancreatic neoplasia development starting from PanIN in both human and mouse models with KRAS mutations [97]. Another study looking at B cell distribution in human PDA demonstrated that B cells retained in tertiary lymphoid tissue gave a survival benefit, which was not seen when B cells were infiltrating into the tumor stroma [99]. Additionally, there are increased levels of B cell activating factor (BAFF) expressed by B cells infiltrating PDA with a correlation with increased EMT-related gene expression in tumor cells [100]. In other solid tumors, B cells have been found to increase tumor invasiveness through secretion of IL-8 [101].

B cells can produce a variety of cytokines and chemokines that have been implicated in immunosuppression. Regulatory B cells (Bregs) secrete IL-10 and TGF-β that induce Treg differentiation via Stat3 as well as M2 macrophage development in different murine cancer models [96, 98, 102]. In other solid tumors, B cells have been implicated in programming myeloid derived suppressor cells (MDSCs) to increase their immunosuppressive activity [98]. Bregs have also been shown to express PD-L1 on their cell surfaces, which can directly inhibit T cells [98]. In PDA models, B cell deficiency has shown to decrease the desmoplastic reaction of tumors [103]. B cells grown in a PDA environment were also shown to encourage Th2 differentiation of CD4 + T cells in a manner dependent on Bruton’s tyrosine kinase [103].

While not yet shown in PDA, it has been shown in other tumors that HIF-1α induces CXCR4 and HIF-2α induces CXCL12 production by B cells [104, 105]. This recruits MDSCs and regulatory T cells (Tregs) to the tumor environment. Interestingly, HIF-1α knockout in the pancreas of mouse PDA showed a large increase in the number of B cells in the tumor microenvironment [106]. The mechanism for this was thought to be increased levels of B cell attractant chemokines such as CXCL13 in the HIF-1α knockout mice PDA models, suggesting that hypoxia helps decrease B cell tumor infiltration [106]. Depletion of B cells via anti-CD20 antibody in the HIF-1α knockout PDA model allowed T cell infiltration into the tumor, but did not change Treg percentage [106].

## Myeloid derived suppressor cells

MDSCs are a progenitor cell type derived from the bone marrow that has recently received increasing attention. These cells can give rise to macrophages, dendritic cells, and granulocytes, among others, and can have a major influence in the immunophenotype of the tumor microenvironment despite their usual low numbers [107]. MDSCs normally circulate in the bloodstream and are drawn to areas of inflammation and ischemia through chemokine molecules such as CXCL12 produced by fibroblasts, as well as growth factors such as granulocyte–macrophage colony stimulating factor (GM-CSF) from tumor cells in PDA [108–110]. Both peripheral blood, bone marrow, and PDA tumors in humans showed MDSC accumulation compared to healthy controls [111, 112]. MDSCs secrete immunosuppressive cytokines such as IL-6, IL-10, and TGF-β, which promote Treg differentiation and inhibit co-stimulatory molecules on antigen presenting cells (APCs) [107, 113]. Macrophage differentiation is also heavily influenced by MDSCs via the production of IL-4, IL-10, and IL-13, which promote an immunosuppressive M2 phenotype via STAT6 [107, 114].

Additional ways that MDSCs exert inhibitory influences on effector T cells is through cell metabolism, although this has primarily been demonstrated in other solid tumors. Via STAT3 and NF-κB pathway activation, MDSCs decrease essential amino acids for T cells, such as tryptophan in breast cancer [115]. l-arginine is depleted via cleavage by arginase-1 and cysteine is accumulated in the cytoplasm due increase uptake and lack of exporter on MDSCs [113, 116]. Metabolites also accumulate to inhibit T cells such as adenosine via up regulation of CD39 and CD73 that cleave ADP and AMP, respectively [79, 107, 117]. MDSCs also nitrate tyrosine residues on T cell receptors that prevent them from accurately recognizing antigens. This occurs via peroxynitrate generation from NO and reactive oxygen species (ROS). In murine PDA models specifically, production of pancreatic adenocarcinoma upregulated factor (PAUF) by tumor cells resulted in increased levels of arginase, NO, and ROS produced by MDSCs [118]. Finally, MDSCs cleave L-selectin due to constitutive expression of ADAM17 on their cell surface, which impairs T cell homing [119].

Lung cancer models have shown increased CD39 and CD73 production by MDSCs in hypoxia [120]. In mouse models of liver tumors, hypoxia increased PD-L1 expression on MDSCs, which had an immunosuppressive effect on T cells [121]. Subsequent blockage of this by PD-L1 inhibition led to decreased IL-10, IL-6, and TGF-β production [121]. Additionally, MDSCs have been shown to increase in number and remain undifferentiated in hepatocellular carcinoma in hypoxic conditions via up regulation of CCL26 and CD391L [122, 123]. Interestingly, HIF-1α stabilization in MDSCs in the lymphoma environment, but not the spleen, supported differentiation into macrophages with increased arginase and NO synthetase levels [124].

## Macrophages

Macrophages are a primary component of the innate immune response, and there has been significant interest in the role of tumor-associated macrophages (TAMs) in the recent years. Macrophages may differentiate from cells in the tumor microenvironment or be recruited via CCL2, CCL5, and CXCL12 [108, 125]. As with most cells in the immune microenvironment, macrophages exist on a spectrum from immunostimulatory to immunosuppressive, which is thought to be adaptive to situations like chronic infections [126]. The milieu of cells in the tumor microenvironment produces cytokines such as IL-4, IL-10, IL-13, and M-CSF that encourage M2 or immunosuppressive phenotype differentiation [127]. Many studies have looked at models for macrophages in a variety of tumors and all have shown that increased TAMs led to decreased survival through various mechanisms [128–131].

Looking at immunosuppressive mechanisms in PDA specifically, macrophages isolated from human PDA have been shown to induce EMT related changes in various cell lines for both M1 and M2 macrophages [132]. Macrophages in PDA also secrete FAP, a serine proteinase, which encourages fibroblasts in the tumor environment to promote tumor angiogenesis and metastasis [87, 133]. Additionally, macrophages are thought to induce PDA cells to produce cytidine deaminase, which metabolizes gemcitabine to promote resistance [134].

M2 macrophages tend to be found in more hypoxic regions of PDA, whereas M1 macrophages tend to be in normoxic regions farther from cancer cells [135, 136]. This spatial arrangement is thought to be due to mechanisms related to IL-6, TGF-β, and M-CSF, as well as semaphorin 3A/neuropilin-1 [137–139]. After migration, semaphorin is then down regulated by HIF-1α, which helps retain M2 macrophages in the hypoxic areas [137, 140]. Interestingly, in bacterial infections, HIF-1α also increases production of IFN-γ which is pro-inflammatory and promotes a more M1 type phenotype, although this was not studied in PDA directly [141]. Secretion of TGF-β and IL-10 from tumor cells and the surrounding environment then promotes macrophage switching to M2 phenotype [108].

TAMs drive significant metabolic changes that influence the microenvironment. In response to hypoxia in a breast cancer model, HIF-1α acts quickly to increase NO via inducible nitric oxide synthetase (iNOS) in macrophages which causes T cell suppression [142]. HIF-2α acts more slowly via increased arginase which decreases the arginine required for NO synthesis and thus counteracts some of the action of HIF-1α; however, the lack of arginine causes more long-term anergy of T cells [142, 143]. Surprisingly, high quantities of NO produced by macrophages in response to HIF-1α can actually lead to tumor suppression and death in early stages [108]. IL-4 can counteract this by up regulating HIF-2α to increase arginase as well as non-HIF mechanisms to increase arginase via NF-κB [142]. In addition to NO regulation, other metabolic pathways such as IDO, which leads to tryptophan depletion, are up regulated in hypoxic macrophages in hepatocellular carcinoma [144].

There are several other proposed mechanisms through which macrophages having an immunosuppressive influence in a hypoxic environment. In a study looking at mouse models of breast, colon, and liver tumors, knockout of HIF-1α and HIF-2α decreased tumor growth, but only HIF-2α knockout lead to decreased expression of macrophage colony-stimulating factor receptor (M-CSFR) and CXCR4 on tumor-infiltrating macrophages [145]. Increased HIF-1α stabilization has also been shown to correlate with increased PD-L1 expression on macrophage cell surfaces [145]. Hypoxia also causes macrophages to produce MMP-7 which can cleave Fas ligand from neighboring cells and protect them from cell-mediated killing [140, 146]. Additionally, increased MMP-2 and MMP-9 have been seen in other solid tumors in the setting of increased tumor cell invasion [136].

## Dendritic cells

Dendritic cells (DCs) are hematopoetic in origin and their main functions are phagocytosis and antigen presentation. Based on the presence or absence of co-stimulatory molecules, DCs can induce pro-inflammatory responses or immune tolerance in other immune cell populations [147]. DCs initially exist in their immature forms, and after exposure to different environmental stimuli, can become more immunostimulatory or immunosuppressive along a spectrum of myeloid forms, which stimulate Th1 response, and plasmacytoid forms, which stimulate a Th2 response [108, 148]. Tumors such as PDA benefit from immature DCs and prevent maturation via production of VEGF, IL-10, IL-6, and GM-CSF, among others [149, 150]. IL-8 also influences DC migration in colon cancer models [151]. Dendritic cells have CXCR1 and CXCR2 on their cell surfaces that binds to IL-8, but the amount of IL-8 did not affect the expression of MHC class II or co-stimulatory molecules such as CD80 and CD86 [151]. Prolonged exposure to IL-8 caused internalization of CXCR1 and CXCR2 prevented further migration towards IL-8 producing tumor cells [151].

In PDA, increased levels of circulating myeloid DCs can be predictive of longer survival after surgical resection [152, 153]. In addition to decreased co-stimulatory molecules such as CD40 and CD80 that prevent T cell activation, DCs in PDA produce a variety of chemokines and cytokines that help support an immunosuppressive environment [148]. DCs can produce CCL22 which recruits immunosuppressive Tregs in response to IL1a and TGF-β [154, 155]. Looking at miRNA in PDA, studies have shown that exosomes from tumor cells can alter cell surface expression of toll-like receptor (TLR) 4 [156]. PAUF is a ligand for TLR4, which can induce the production of pro-inflammatory TNF-α and IL-12 by DCs [156, 157]. Additionally, Smad4, a transcription factor that mediates TGF-β transduction, is repressed in DCs in the PDA environment through miRNA which prevents their antigen presentation and differentiation [158].

There are conflicting reports on the effects of hypoxia on DCs. In some reports, hypoxia increases the ability of DCs to interact with cytotoxic T cells [159]. Hypoxia has been shown to decrease circulating plasmacytoid DCs with corresponding increase in TNF-α and IL-6, although increase in CXCL12 could signify tissue migration [160]. One experiment demonstrated that DCs without HIF-1α had less CD278 on their cell surface, and the T cells in co-culture produced less granzyme B mRNA [161]. This has been shown to be due to the PI3K/Akt pathways [162]. It is not certain if DCs have decreased migration and phagocytic capabilities in a hypoxic environment [163, 164]. There does seem to be increased osteopontin secreted by hypoxic DCs in a breast mouse model that encourage tumor migration [165]. Hypoxia encourages the Th2 phenotype via increase in CD44 as well as adenosine receptor A2b although this has not been verified in PDA specifically [166, 167]. Additionally, PD-L1 expression on DC membranes is increased due to HIF-1α in hypoxia [121, 145]. Some immature DCs with prolonged exposure to hypoxia can actually be induced to undergo apoptosis via up regulation of BNIP3 and BAX [168].

## Helper and regulatory T cells

CD4^+^ T cells have several different subtypes that result from terminal differentiation of naive progenitor cells, although there is some overlap between these classifications. The most commonly researched in PDA are T helper (Th) 1, Th2, Treg, and Th17 [169]. Th1 cells are pro-inflammatory and express IFN-γ to promote APCs and prime CD8^+^ cells, while Th2 cells may encourage tumor growth through IL-5 production, although they do recruit eosinophils through IL-4 and IL-13 [170]. Tregs are identified by FOXP3 gene expression and are predominately immunosuppressive through TGF-β production and repression of effector T cell proliferation [170]. Less clear is the role of Th17 cells that have been studied in autoimmune disease and may be associated with prolonged survival in some cancers [171, 172]. In addition to differing roles, CD4^+^ subtypes are also located differently in the PDA microenvironment. While the percentage of CD4^+^ cells that were Th1 remained stable throughout the tumor, tumor periphery, and healthy pancreas, Th2 and Treg cells were more likely to be in the central tumor, whereas Th17 cells were more likely to be in the healthy pancreas [169]. Another study showed that Tregs tend to accumulate early in the malignant process, even in PanIN mouse models, but primarily remain in peritumoral lymph nodes [173].

Due to poor response of PDA to immunomodulating therapies such as checkpoint inhibition, a significant focus has been placed on the immunosuppressive Tregs. CCL5 is produced by PDA which encourages migration of Tregs into the tumor microenvironment due to their CCR5 expression [174]. Treg migration into PDA is also encouraged by L1CAM expression on tumor cells [175]. Tregs in PDA express CTLA-4, which competes for co-stimulatory ligands CD80 and CD86 and prevents CD28 binding necessary for effector T cell activation [173]. Many Tregs also express CD25, which acts as an IL-2 receptor and contributes to FOXP3 expression in some Tregs [173, 176]. Overall, increased ratios of Treg:effector T cells is associated with tumor progression and worse outcome [12].

Hypoxia influences immunosuppression as well as subtype differentiation of CD4^+^ cells. Activation of the T cell receptor actually increases HIF-1α downstream effects via PI3K/mTOR and protein kinase C mechanisms through stabilization of HIF-1α even in the absence of hypoxia [140, 177]. HIF-1α promotes differentiation into FOXP3^+^ cells via increased gene transcription [178]. If sufficient TGF-β is also present, then these cells become Tregs, but combined IL-6 and HIF-1α instead promotes Th17 cells [179, 180]. Addition of IL-6 actually counteracts the effects of hypoxia on Treg proliferation with IL-1 having a more moderate counteractive effect [177, 179]. These mechanisms are thought to be mediated by STAT3, which also decreases IFN-γ production and decreases Th1 phenotype markers [177, 180]. Interestingly, HIF-1α can also bind to the FOXP3 protein to promote its degradation which can also promote Th17 and other pro-inflammatory phenotypes [178]. HIF-2α, however, does not promotes FOXP3 transcription in murine models of inflammation [179]. Treg numbers are further increased in hypoxia due to CCL28 production by tumor cells that increases Treg chemotaxis [181]. Hypoxia does not appear to affect levels of co-stimulatory molecules such as CD23 and CTLA-4 in CD4 + cells [179].

## Effector T cells

Effector or CD8^+^ T cells are the primary cytotoxic agents of the adaptive immune systems. Most of the immunosuppressive mechanisms discussed above involve preventing effector T cells from undergoing the steps required to induce apoptosis in tumor cells: [1] migration of a naive CD8^+^ T cell into the area of the tumor, [2] antigen-presentation to the CD8^+^ cell between the T cell receptor (TCR) and MHC class I molecule with appropriate co-stimulation (via cytokines from CD4^+^ cells or CD28 and CD80/B7-1 or CD86/B7-2 on the APC), [3] clonal expansion of CD8^+^ cells, [4] recognition of the antigen again on the tumor cell, and [5] initiation of cytotoxic process with production and release of perforin, granzyme, and granulysin which trigger the caspase cascade in tumor cells [182, 183]. Alternatively, CD8^+^ cells have Fas ligand/CD95L on their cell surface that can bind Fas on target cells, which then promotes procaspases in the target cell, but this is rarely expressed in tumor cells [182]. The above describes more common protein-recognizing αβ CD8^+^ cells; however, there is a separate population of γδ CD8^+^ cells that recognize lipid antigens and do not require the same antigen presentation steps to become activated. Indeed, γδ CD8^+^ cells are being studied as well in immunotherapy for PDA [184].

Among the more common αβ CD8^+^ cells, there have been both stimulatory and inhibitory effects of hypoxia. HIF-1α is essential to the expression of the co-stimulatory molecule CD137 on effector T cells in several solid tumors, although this has not been confirmed in PDA [185]. Also, granzyme B production is increased in hypoxia, leading to increased lytic capacity of T cells in more mildly hypoxic compared to atmospheric oxygen [186–188]. Negative effects include decreased effector T cell migration into the tumor in hypoxia. Poorly formed vasculature forms in the hypoxic PDA environment, and in combination with IL-10 down regulating integrins such as αLβ2 on the vascular endothelium, T cell extravasation is diminished [140, 189]. HIF-1α also decreases production of the pro-inflammatory cytokines IL-2 and IFN-γ by CD8^+^ cells, even when stabilized under normoxic conditions [177, 186]. ROS resulting from hypoxia can have immunosuppressive and even lethal effects on T cells. Superoxide is an ROS produced in mitochondria from activation of STAT3 and NADPH, which can then activate the caspase cascade and cause T cell apoptosis [188]. Reactive nitrogen species such as peroxynitrite, are generated from ROS, and can prevent the binding of molecules to T cell receptors through nitration of receptor amino acids [190].

More recent studies have also been examining the changes in metabolism between naive, effector, and memory CD8^+^ cell populations. Upon activation by binding at the TCR and the appropriate co-receptors, CD8^+^ cells preferentially use glycolysis similar to APCs after binding of TLRs or other pathogen-receptor-recognition pathways [191, 192]. The focus on glycolysis is thought to be maintained in part due to HIF-1α stabilization even in the absence of hypoxia [193]. However, when glucose becomes scarce in the tumor microenvironment due to uptake by cancer cells, CD8^+^ cells switch to oxidative phosphorylation and have increased PD-1 expression [193]. The hypoxic tumor environment prevents the successful transition to oxidative phosphorylation, which can lead to decreased proliferation and increased LAG3 expression in melanoma mouse models [193]. Multiple studies have shown that in hypoxic and glucose deficient states, CD8^+^ cells switch to fatty acid oxidation via PPAR-α pathway signaling, and that this transition is necessary to prevent T cell exhaustion in this environment [192, 193]. Interestingly, many are studying PPAR antagonists in solid tumors to disrupt similar pathways in cancer cells, but may have negative effects on the increase of fatty acid oxidation in T cells [194].

## Future directions

Remarkable progress has been made in the field of immunotherapy. Particularly in melanoma and lung cancers, patients enjoy longer survival, and in rare cases, complete remission in response to immunotherapy [195, 196]. Immunotherapy for pancreatic cancer has not yet shown the same degree of success, but many have chronicled the progress thus far in detailed reviews [197–200]. Single-agent immunotherapy clinical trials in human PDA have particularly been ineffective, thought in part to be due to decreased PD-1/PD-L1 expression compared to other tumors, but many ongoing studies are examining combinations, including with cytotoxic chemotherapy and radiation [75, 201]. Novel therapies including DC vaccines, chimeric antigen receptor T cells, and miRNA inhibitors are being developed, to name a few [202]. It is likely that effective treatment will take a combination of these therapies and that the heterogeneity of PDA will prevent a one-size-fits-all treatment model.

During the testing of new therapies it is important to replicate the conditions inside the human tumor as much as possible. Hypoxia, as described above, has a significant influence on the immune response to PDA, yet most new therapies are tested in cell cultures in atmospheric oxygen environments. Particularly with T cells, atmospheric oxygen compared to physiologic oxygen can cause lower intracellular NO and decreased CD69, which can cause increased T cell proliferation [203]. Further studies also need to be done regarding regulation of T cell metabolism in hypoxia, including promotion of fatty acid oxidation in glucose and oxygen poor environments. Mouse models, while they do have hypoxic tumor environments, also have limitations. Both genetically engineered and xenograft PDA mouse models do not have as robust a T cell infiltrate as human tumors, despite having more circulating lymphocytes [12, 84, 204]. Other differences between mice and humans include regulators of iNOS in macrophages, induction of Th1 responses in T cells in response to IFN-α, and expression of T cell co-stimulatory molecules such as CD28 [204]. Trends lately have been to “de-sterilize” laboratory animal environments to help their immune systems better reflect those in humans, but barriers remain [205, 206]. Newer organoid models have also been developed that enable a 3-D structure, heterogeneity, and interactions between cell types [207]. Advantages of the organoid model include a longer lifespan than a tumor slice culture model and the ability to expand and use in xenografts unlike a tumor slice culture model [208].

Targeting of the hypoxic environment in solid tumors has also been attempted (Table 1). The most basic of these ideas is to create a tumor microenvironment with increased oxygen or ROS to sensitive the cancer cells to radiation and other therapies, however others argue that anti-oxidants such as N-acetylcysteine that decrease ROS may actually decrease ROS and therefore downstream effects of EMT and immunosuppression [209]. Other methods include pro-drugs that become activated in hypoxia or drugs that hone in on HIF-1α active cells are being developed [20, 210, 211]. Evofosfamide (TH-302), a mustard-based derivative, is a cytotoxic pro-drug that is converted to an active metabolite in hypoxic conditions [211]. Evofosfamide has been used to decrease resistance to radiotherapy in pancreatic cancer, but a recent clinical trial for non-small cell lung cancer using this drug in combination with tarloxotonib, a hypoxia-activated tyrosine kinase inhibitor, was stopped early due to futility [211, 212]. Another drug developed over a decade ago is POP33, a fusion protein that consists of a transduction domain to deliver the drug into cells, a HIF-1α dependent stabilization domain, and a cleaved caspase pro-enzyme [210]. While this showed promise in a mouse model of PDA, there has yet to be a successful human application. While no direct HIF inhibitors have been used in clinical trials for pancreatic adenocarcinoma, therapies that target heat shock protein (HSP) 90 have shown to also lead to HIF degradation and are currently being tested [213, 214].

**Table 1:** Therapies targeting hypoxia or hypoxic downstream effects in pancreatic adenocarcinoma

Category	Mechanism	Drug(s)	Clinical trials in pancreatic adenocarcinoma
Simulates increased oxygen levels	Electron affinity increases free radical formation	Misonidazole	Pre-clinical, non-pancreatic cancers
	Promote oxygen dissociation from hemoglobin	OXY111A	NCT02528526 (unknown)
	Small molecule enzyme mimetic that converts superoxide to hydrogen peroxide and oxygen	GC4419	NCT03340974 (recruiting)
Hypoxia-activated pro-drug	Converted to active cytotoxic drug via cellular reductases	Evofosfamide (TH-302)—Mustard Apaziquone (E09)—Mitomycin C Tirapazamine—Free radical	NCT01746979 (completed), NCT02402062 (active), NCT00743379 (completed), NCT02047500 (terminated)
	Hypoxia activated topoisomerase	Banoxantrone/AQ4N	NCT00090727 (unknown)
Hypoxia-targeting fusion protein	Protein-transduction domain, oxygen dependent degradation domain similar to HIF-1α, and cleaved caspase 3 pro-enzyme or other cytotoxic element	POP33	Pre-clinical
Gene-Directed Enzymatic Pro-drug Therapy (GDEPT)	Viral vectors transfer heterologous gene to tumor cells and hypoxia response element in promoter causes increased transcription of target gene in hypoxia to convert pro-drug to active form	Vectors (retroviruses) containing suicide genes such as Herpes simplex virus thymidine kinase, cytosine deaminase, or cytochrome P450	Pre-clinical
Direct HIF inhibition	Binds to HIF-1α and/or HIF-2α and inhibits dimerization and transcription	Acriflavine PT2385	Pre-clinical, Non-pancreatic cancers
	Inhibits HSP 90 leading to HIF-1α degradation	XL888 Geldanamycin/tanespimycin Luminespib (AUY922)	NCT03095781 (recruiting) NCT00577889 (completed) NCT01484860 (terminated)
Inhibition of major pathways influenced by HIF	STAT3 inhibitor	Napabucasin (BBI608)—small molecule AZD9150—antisense oligonucleotide	NCT02231723 (active) NCT02983578 (recruiting)
	Notch inhibition via gamma-secretase inhibitor	MK-0752 Demcizumab RO4929097	NCT01098344 (completed), NCT02289898 (completed), NCT01145456 (completed), NCT01131234 (completed), NCT01232829 (completed)
	Notch inhibition via binding delta-like ligand 4	TGR-1202	NCT02574663 (active)
	Notch inhibition via antibody targeting Notch 2/3 receptors	Tarextumab (OMP-59R5)	NCT01647828 (completed)
	PI3K inhibition via small molecule binding	Alpelisib (BYL719) Buparlisib (BKM120) Rigosertib (ON 01910.Na) Gedatolisib LY3023414 Dactolisib (BEZ235)	NCT02077933 (active), NCT02155088 (active) NCT01571024 (completed), NCT01360853 (completed), NCT03065062 (recruiting), NCT02981342 (active) NCT01658436 (completed), NCT01155453 (completed), NCT01337765 (completed)
	Hedgehog signaling pathway inhibition via signal transducer smoothened inhibition by small molecule	Sonidegib (LDE-225) Vismodegib (GCD-0449) Saridegib (IPI-926)	NCT01487785 (completed), NCT01195415 (completed), NCT01064622 (completed), NCT00878163 (active), NCT01537107 (suspended), NCT01383538 (completed)
Inhibition of metabolic changes associated with hypoxia	Shifts cell metabolism from glycolysis to oxidate phosphorylation	BPM31510—liposomal CoQ10	NCT02650804 (recruiting)
	Indolamine 2,3 dioxygenase-1 inhibitor to prevent trypthophan depletion	Epacadostat	NCT03006302 (recruiting), NCT03432676 (withdrawn)
	Inhibition of CD73 to prevent adenosine accumulation	CPI-006—anti-CD73 humanized antibody Oleclumab (MEDI9447)—anti-CD73 monoclonal antibody	NCT03454451 (recruiting) NCT03611556 (recruiting)
	Inhibition of A2A adenosine receptor to prevent adenosine binding on lymphocytes	NIR178—small molecule CPI-444—small molecule	NCT03207867 (recruiting) NCT03454451 (recruiting)
	Interference with MMP-9 upregulation	Zoledronic acid—decrease MMP-9 production by myeloid derived cells Andecaliximab—Anti-MMP-9 monoclonal antibody	NCT00892242 (terminated) NCT01803282 (active)
Inhibition of cytokines upregulated by hypoxia	TGF-β inhibition via prevention of binding to receptor or signal transduction	Galunisertib—TGFbR1 Vactosertib—binds TGFbR1 M7824—fusion protein binds TGFbR2 NIS793—monoclonal antibody to TGFb	NCT02734160 (active), NCT02154646 (completed), NCT01373164 (completed), NCT03666832 (not yet recruiting), NCT03451773 (suspended), NCT02947165 (recruiting)
	TGF-β inhibition via TGF-β2 antisense oligonucleotide	AP 12009	NCT00844064 (completed)
	IL-6 inhibition	Tocilizumab—anti-IL-6 receptor Siltuximab—anti-IL-6	NCT02767557 (recruiting) NCT00841191 (completed)
Inhibition of chemokines upregulated by hypoxia	Prevention of PD-1/PD-L1 checkpoint inhibition via anti- PD-L1 antibodies	Durvalumab Avelumab M7824 LY3300054 Atezolizumab	NCT03257761 (recruiting), NCT03376659 (recruiting), NCT02403271 (completed), NCT03245541 (recruiting), NCT03572400 (recruiting), NCT02868632 (recruiting), NCT02639026 (recruiting), NCT02734160 (active), NCT03344172 (recruiting), NCT03637491 (recruiting), NCT03829501 (recruiting)
	Inhibition of Treg migration via CCR2/CCR5 antagonist	BMS-813160—small molecule	NCT03184870 (recruiting), NCT03767582 (not yet recruiting)
	Inhibition of MDSC and Treg recruitment via decreased CXCR4 inhibition	BL-8040—small molecule Plerixafor—small molecule	NCT02907099 (recruiting), NCT02826486 (unknown), NCT03277209 (active), NCT02179970 (completed)
	Inhibition of MDSC and Treg recruitment via decreased CXCL12(SDF-1) inhibition	Olaptesed—small molecule	NCT03168139 (active)

HIF, hypoxia-inducible factor; STAT, signal transducer and activator of transcription; PI3K, phosphoinositide 3-kinase; HSP, heat shock protein; MMP, matrix metalloproteinases; TGF, transforming growth factor; PD, programmed cell death; MDSC, myeloid derived suppressor cell; Treg, regulatory T cell; SDF, stromal-derived factor

Major limitations of targeting HIF with inhibitors are the rapid degradation of the molecule, as well as the highly conserved nature of the transcription pathways and potential for negative systemic effects [215, 216]. Indeed several clinical trials have examined targeting STAT3, Notch, PI3K, and Hh pathways in PDA without strongly favorable results [217–222]. Several therapies have been developed to target the more downstream effects of hypoxia, however. Countering Warburg metabolism is one strategy, as is encouraging fatty acid oxidation in T cells. CD73 upregulation and subsequent accumulation of immunosuppressive adenosine has been targeted via anti-CD73 antibodies, as well as anti-A2A adenosine receptor inhibition, which is present on T cells [78, 120]. Preventing hypoxic upregulation of MMP-9, used in cancer cell and MDSC migration, via zoledronic acid is also being studied as combination therapy in PDA [223, 224]. Cytokine and chemokines that are upregulated in hypoxia have also been targeted. Immunosuppressive TGF-β and IL-6 are the targets of several clinical trials in PDA [225–228]. Increased PD-L1 expression is seen on on carcinoma cells in PDA as well as MDSCs and macrophages; this has been targeted with both anti-PD-L1 antibodies and inhibition of pyruvate kinase M2, another molecule that binds in the PD-L1 promoter [229]. Many clinical trials treating PDA that are actively recruiting involve PD-L1 inhibition (Table 1). The CXCR4-CXCL12 axis as well as the CCR5 chemokine with its multiple receptors are also being targeted in PDA [84, 230]. It is possible that combining more developed immunotherapy such as checkpoint and/or chemokine inhibitors with hypoxia targeting may finally overcome the severe immunosuppressive milieu in PDA.

## Conclusions

Hypoxia exists in PDA in a heterogeneous manner, and the complex immunosuppressive environment in PDA is exacerbated in hypoxic conditions. Immunotherapy in PDA is not yet successful, likely due to the numerous immunosuppressive pathways upregulated in hypoxia. Tumor heterogeneity will prevent a one-size fits all approach for traditional chemoradiotherapies as well as immunotherapies, but it is important to test in conditions that most resemble the hypoxic human tumor microenvironment.

## Abbreviations

PDA: Pancreatic ductal adenocarcinoma
TIL: tumor-infiltrating lymphocytes
HIF: hypoxia inducible factor
vHL: von Hippel-Lindau protein
IDO: indoleamine 2,3 dioxygenase
EMT: epithelial to mesenchymal transition
MMPs: matrix metalloproteinases
ECM: extracellular matrix
VEGF: vascular endothelial growth factor
TNF: tumor necrosis factor
TRAIL: TNF related apoptosis inducing ligand
MHC: major histocompatibility complex
NO: nitric oxide
HLA-G: human leukocyte antigen G
PD-L1: programmed death ligand 1
MDSCs: myeloid derived suppressor cells
PSCs: pancreatic stellate cells
aSMA: alpha smooth muscle actin
PanIN: pancreatic intraductal neoplasms
FAP: fibroblast activating protein
BAFF: B cell activating factor
Bregs: Regulatory B cells
Tregs: Regulatory T cells
GM-CSF: granulocyte–macrophage colony stimulating factor
APCs: antigen presenting cells
PAUF: pancreatic adenocarcinoma upregulated factor
TAMs: tumor-associated macrophages
iNOS: inducible nitric oxide synthetase
M-CSFR: macrophage colony-stimulating factor receptor
DCs: dendritic cells
TLR: toll-like receptor
TCR: T cell receptor
HSP: heat shock protein
LAG3: lymphocyte activation gene 3
